# Design of a New 1D Halbach Magnet Array with Good Sinusoidal Magnetic Field by Analyzing the Curved Surface

**DOI:** 10.3390/s21072522

**Published:** 2021-04-04

**Authors:** Guangdou Liu, Shiqin Hou, Xingping Xu, Wensheng Xiao

**Affiliations:** College of Mechanical and Electronic Engineering, China University of Petroleum, Qingdao 266580, China; gdliu@upc.edu.cn (G.L.); z19040065@upc.edu.cn (S.H.); web_tom@163.com (X.X.)

**Keywords:** Halbach magnet array, curved surface, good sinusoidal magnetic field, linear motor, planar motor

## Abstract

In the linear and planar motors, the 1D Halbach magnet array is extensively used. The sinusoidal property of the magnetic field deteriorates by analyzing the magnetic field at a small air gap. Therefore, a new 1D Halbach magnet array is proposed, in which the permanent magnet with a curved surface is applied. Based on the superposition of principle and Fourier series, the magnetic flux density distribution is derived. The optimized curved surface is obtained and fitted by a polynomial. The sinusoidal magnetic field is verified by comparing it with the magnetic flux density of the finite element model. Through the analysis of different dimensions of the permanent magnet array, the optimization result has good applicability. The force ripple can be significantly reduced by the new magnet array. The effect on the mass and air gap is investigated compared with a conventional magnet array with rectangular permanent magnets. In conclusion, the new magnet array design has the scalability to be extended to various sizes of motor and is especially suitable for small air gap applications.

## 1. Introduction

Linear and planar motors can directly convert electric energy into linear motion mechanical energy without any transmission device of intermediate conversion mechanism [1,2]. They have the advantages of compact structure, high transmission stiffness, fast dynamic response, and high positioning accuracy due to the absence of mechanical conversion parts, and are extensively used in various fields, such as logistics delivery systems, computer numerical control (CNC) machine systems, lithography, and magnetically levitated train systems. The magnetic field on one side of the 1D Halbach permanent magnet array is significantly enhanced due to the arrangement of permanent magnets and has good sinusoidal characteristics. Therefore, the 1D Halbach magnet array is widely applied in linear, planar, and other motors [3,4,5,6,7,8].

Many other researchers have studied linear or planar motors by applying the 1D Halbach magnet array. Won-Jong Kim et al. [9,10] proposed the planar magnetic levitation device, which is composed of four permanent magnet linear motors. The 1D Halbach magnet array with rectangular magnets is used and the magnetic field is represented by Fourier series expansion and the complex theory. M Lee et al. [11] replaced the rectangular magnets with trapezoidal magnets in the double-sided linear motor to produce more force. The magnetic flux density distribution was obtained by adding the magnetic flux density of each permanent magnet. Chen Jun-Wei et al. [12] proposed a new Halbach magnetic array by applying the permanent magnet with a sinusoidal edge. The larger flux density and smaller harmonic distortion can be obtained by the magnet array in a linear motor. The magnetic flux density was derived by generalized blending function mapping and superposition. Irfan-Ur-Rab Usman et al. [13] developed a 6 degree of freedom (DOF) planar levitating synchronous motor. It consists of four linear motors with 1D magnet arrays. The coil is made into a printed circuit board. The force fluctuation can be reduced by designing proper separation and spacing magnet arrays. Rui Chen [14] analyzed the M-Magnet array with a magnetization axis in a 45° direction relative to its side surfaces, instead of 0° or 90° magnetization pieces used in a conventional Halbach array. Then, a novel hybrid array based on the M-Magnet array is presented which can attenuate the 6th force ripple. A. Boduroglu et al. [15] proposed a new, skewed magnet arrangement for the linear motor. By comparing the conventional magnet arrangement, the permanent magnet with asymmetric V shape arrangement can reduce the force ripple and has little effect on the average force.

**Figure 1:**
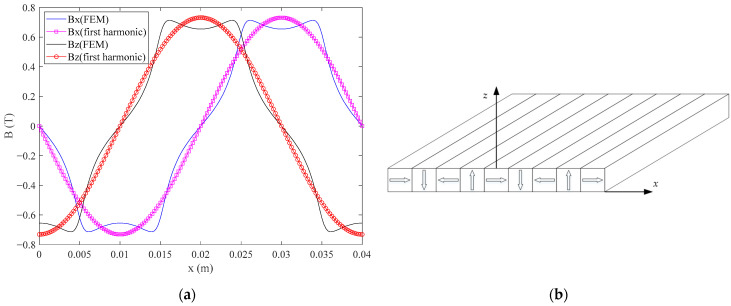
(**a**) The first harmonic and FEM of magnetic flux density; (**b**) the conventional magnet array with rectangular magnets.

According to the above analysis, in order to improve the performance of the permanent magnet array, a variety of studies have been carried out, such as high force, small harmonic distortion, and low force ripple. In the linear and planar motor, the air gap is very small between the permanent magnets and coils in order to get higher thrust. However, the sinusoidal property of the magnetic field deteriorates when the air gap is small. This can be seen in Figure 1, obtained by the conventional 1D Halbach magnet array with rectangular magnets when the air gap is 1 mm. In the real-time control, the first harmonic of the magnetic flux density is usually used to calculate the force, which will cause the force ripple due to the error with the actual magnetic flux density. 

It is found that the magnetic flux density has poor sinusoidal property and relatively large error compared with the first harmonic. As we know, one permanent magnet can be regarded as a superposition of countless small pieces according to the superposition principle, so does the magnetic flux density of the permanent magnet. Therefore, the main cause of poor waveform is that the rectangular permanent magnet is used, such as in [1,2,3,4,5,6,7,8,9,10,13,14]. Though the trapezoidal magnet [11], the sinusoidal-edged magnet [12], and the skewed magnet [15] are applied in the motors, the top and bottom surfaces of permanent magnets are flat and the sinusoidal property of the magnetic field also deteriorates in the small air gap. Therefore, the paper is analyzed from a new perspective. The surface shape of the permanent magnet is designed to be curved instead of flat so that the waveform of magnetic flux density can be improved.

In order to obtain the magnetic flux density with good sinusoidal characteristics, the permanent magnet is divided into small pieces and the heights of the small pieces are designed. The design of a permanent magnet is a constrained nonlinear multivariable optimization problem. The appropriate optimization algorithm [16,17,18,19,20] should be chosen to realize the design of the new magnet array. In terms of numerical effect and stability, the sequential quadratic programming (SQP) algorithm is considered to be one of the most effective methods to solve nonlinear constrained optimization. Therefore, the heights of the small pieces are optimized by SQP. The shape of the curved surface is obtained by optimization results and is fitted by a least squares method [21,22]. Consequently, a new 1D Halbach magnet array with a curved surface is proposed. 

## 2. Modeling of the New Magnet Array

The magnetic flux density is modeled in this section. The new magnet array is proposed based on the conventional magnet array with rectangular magnets. The permanent magnet is designed to obtain a good sinusoidal magnetic field. Therefore, the analysis steps are as follows. Firstly, the permanent magnet is divided into small pieces. Then the height of each small piece is independently set. Finally, the magnetic flux density is derived based on the Fourier series. 

**Figure 2:**
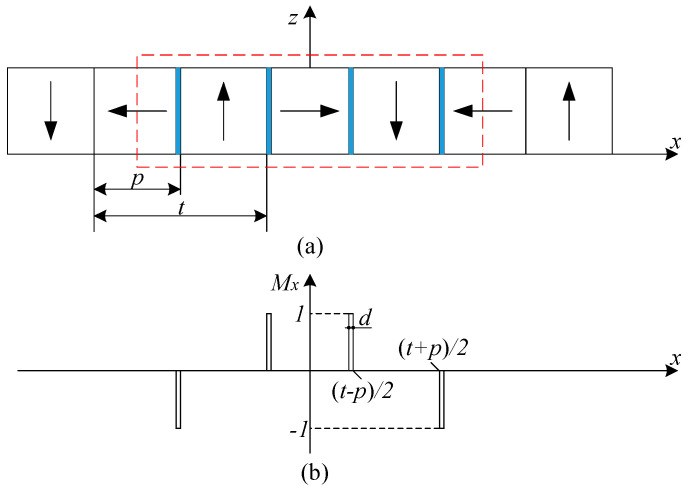
The magnet array and magnetization distribution: (**a**) cross-section of the magnet array; (**b**) *M_x_* projection distribution.

Figure 2 shows the cross-section of the magnet array and the *M_x_* projection distribution for small pieces of permanent magnets in the *x* magnetization direction. The magnetization direction is denoted by the arrow. The direction of the arrow is from the *S* pole to the *N* pole. Each small piece of permanent magnet can be seen as one rectangular magnet and is indicated by the blue bar. 

On the basis of the periodic and symmetry characteristics of the magnet array, all the permanent magnets are divided into the same number of small pieces. The side length of each piece is the same. For each permanent magnet, the axis of symmetry is in the middle and both sides have a symmetrical distribution. Therefore, there are four small pieces with the same heights in one period, and these pieces can be regarded as one group shown in Figure 2. If the number of all small pieces of one permanent magnet is 2*n*, where *n* is an integer, the number of the small pieces of half of one permanent magnet is *n*. From this, the permanent magnets magnetized in the *x*-direction of the magnet array are composed of *n* groups of pieces, and so are the permanent magnets magnetized in the *z*-direction.

The *M_x_* of magnet array is modeled based on the Fourier series and expressed as

(1) $$M_{x}=M\sum_{k=1}^{\infty }\sum_{i=1}^{n}a\left(i,k\right)\cos\left(k\omega x\right),$$

where *M* = *B_r_*/*μ*_0_, *ω* = *π*/*t*, *μ*_0_ is the permeability of vacuum, *t* is the pole pitch, *k* is the harmonic numbers, *a*(*i*,*k*) is projection distribution coefficient, *B_r_* is the remanence of the permanent magnet,

(2) a(i,k)=8kπsin(kωd/2)sin(kπ/2)sin(kω(p+(2i−1)d)/2),

(3) $$d=\frac{p}{2n},$$

where *d* is the side length of the small piece, and *p* is the side length of the permanent magnet.

**Figure 3:**
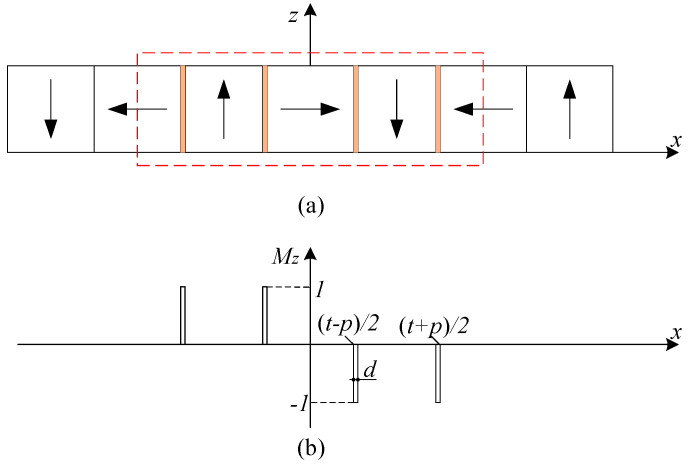
The magnet array and magnetization distribution: (**a**) cross-section of the magnet array; (**b**) *M_z_* projection distribution.

The magnetization vector of the magnet array consists of *M_x_* and *M_z_* components. The *M_z_* component also can be modeled by using the Fourier series. Figure 3 shows the *M_z_* projection distribution of the permanent magnets in the *z* magnetization direction.

The expression of *M_z_* is

(4) $$M_{z}=M\sum_{k=1}^{\infty }\sum_{i=1}^{n}b\left(k,i\right)\sin\left(k\omega x\right),$$

where *b*(*i*,*k*) is the projection distribution coefficient,

(5) b(i,k)=−8kπsin(kωd/2)sin(kπ/2)cos(kω(p−(2i−1)d)/2).

The magnetization vector of the magnet array is obtained and expressed as

(6) $$\vec{M}=\left[\begin{array}{c} M_{x}\\ M_{z} \end{array}\right]=M\sum_{k=1}^{\infty }\sum_{i=1}^{n}\left[\begin{array}{c} a\left(i,k\right)\cos\left(k\omega x\right)\\ b\left(i,k\right)\sin\left(k\omega x\right) \end{array}\right],$$

There are three regions of the magnetic field in space. From top to bottom, they are air, permanent magnet array, and air. The magnetic scalar potential method is applied to solve the magnetic flux density because there is no conduction current.

The boundary conditions of the interface can be derived from Maxwell’s equations. So the magnetic field problem comes down to solve the Poisson equation of magnetic scalar potential [9]. The magnetic flux density is obtained by using the variable separation method. For the region below the magnet array, it is expressed as

(7) $$\vec{B}=\left[\begin{array}{c} B_{x}\\ B_{z} \end{array}\right]=-\mu_{0}\omega \sum_{k=1}^{\infty }Ke^{\lambda z}k\left[\begin{array}{c} \cos\left(k\omega x\right)\\ \sin\left(k\omega x\right) \end{array}\right],$$

where *h_t_*(*i*) and *h_b_*(*i*) are the position of the top and bottom surfaces of each piece of the permanent magnet, respectively,

(8) $$K\left(\mu_{r}=1\right)=\frac{B_{r}}{2\mu_{0}\lambda }\sum_{i=1}^{n}\left(e^{-\lambda h_{t}\left(i\right)}-e^{-\lambda h_{b}\left(i\right)}\right)\left(b\left(i,k\right)-a\left(i,k\right)\right),$$

(9) λ=kω,

The relative permeability for permanent magnet *μ_r_* is assumed as 1.0. Because high-quality sintered NdFeB permanent magnets (*μ_r_* = 1.03~1.05) will be used, the error due to this assumption can be neglected.

## 3. Design of New Magnet Array

### 3.1. Optimization

The main purpose is to obtain a good sinusoidal magnetic field, which is to make the actual magnetic flux density consistent with the first harmonic of the magnetic flux density. As a result, the actual magnetic field has good sinusoidal characteristics, and the expression can be simplified as that of the first harmonic. 

In the real-time control of electrical machines, the first harmonic of the magnetic flux density is usually used to calculate the force. The new magnet array is designed based on the conventional magnet array, and the shape of the surface is optimized. Therefore, the first harmonic of magnetic flux density of the conventional magnet array is chosen in the optimization. 

The optimization is realized by reducing the higher harmonics. The shape of the curved surface is obtained by optimizing the height of small pieces. Generally, the main performance of the motors is reflected by the horizontal thrust. It is produced by the *z* component of the magnetic flux density. So the minimization of the higher harmonics of the *z* component is chosen to be the objective.

For the conventional magnet array, the first harmonic of the magnetic flux density can be obtained when *n* takes 1 and expressed as

(10) $${\vec{B}}_{1}=\left[\begin{array}{c} B_{x1}\\ B_{z1} \end{array}\right]=-\mu_{0}\omega K_{1}e^{\omega z}\left[\begin{array}{c} \cos\left(\omega x\right)\\ \sin\left(\omega x\right) \end{array}\right],$$

where *B*_*x*1_ and *B*_*z*1_ are the first harmonic of *x* and *z* component of magnetic flux density, respectively, *K*_1_ is the coefficient, *m_h_* is the height of the permanent magnet, *m_t_* and *m_b_* are the position of the top and bottom surfaces of the rectangular permanent magnet, respectively,

(11) $$K_{1}=\frac{B_{r}}{2\mu_{0}\omega }\left(e^{-\omega m_{t}}-e^{-\omega m_{b}}\right)\sum_{i=1}^{1}\left(b\left(i,k\right)-a\left(i,k\right)\right),$$

(12) $$m_{h}=m_{t}-m_{b}.$$

In order to determine the harmonic numbers for optimizing, we take the *B_r_*, *t*, *p,* and *m_h_* parameters of the permanent magnet as 1.2 T, 20 mm, 10 mm, and 10 mm for analysis, respectively. The maximum of the magnetic flux density at 2 mm below the *x*-axis is about 1.677 × 10^−5^ T when *k* = 25, which is much less than the geomagnetic field (about 6 × 10^−5^ T). The harmonic components can be ignored in optimization if the harmonic numbers, *k*, are more than 25.

Therefore, the approximate expression of the magnetic flux density can be represented by a certain number of harmonics. The *z* component is given by

(13) $$B_{z}^{m}=-\mu_{0}\omega \sum_{k=1}^{m}Ke^{\lambda z}k\sin\left(k\omega x\right),$$

where *m* = 25.

The higher harmonic components of the new magnet array can be evaluated by

(14) $$B_{zh}=B_{z}^{25}-B_{z1}.$$

According to the periodicity of the magnetic field, the region of half period is chosen. The region is placed 2 mm below the *x*-axis and divided into 41 points. The objective function is given according to Equations (10) and (14)

(15) $$f(h_{t}\left(i\right),h_{p}\left(i\right))=\left|\sum_{m=1}^{41}B_{zh}\left(x_{l},z_{l}\right)/\sum_{m=1}^{41}B_{z1}\left(x_{l},z_{l}\right)\right|\times 100\%,$$

where *x_l_* = 0.025(*m* − 1)*t* and *z_l_* = −0.002 are the coordinate values.

From the objective function, Equation (15), the reduction of higher harmonics is a constrained nonlinear multivariable optimization problem. The sequential quadratic programming (SQP) algorithm has the advantages of good convergence, high computational efficiency, and strong boundary searchability, which is chosen to realize the optimization. In order to avoid the singular points and obtain better optimization results, the number of pieces of half of one permanent magnet is set to five after analysis. For the whole permanent magnet, the total number of small pieces is 10. It is enough to exhibit well the shape of the curved surface of the permanent magnet. 

The optimization parameters and variables are shown in Table 1 and Table 2, respectively. Considering the assembly of the permanent magnets, the positions of the top surface of each piece (*h_t_*(1), *h_t_*(2), …, *h_t_*(5)) remain the same, and the optimization variables are the positions of the bottom surface of each piece of half of one permanent magnet (*h_p_*(1), *h_p_*(2), …, *h_p_*(5)). The ‘constraints’ in Table 2 define the range of optimization variables. Since the constraints of the five optimization variables are the same, they are represented by *h_p_*(1)~*h_p_*(5). Considering the optimization parameters, the upper bound of optimization variables does not exceed the position of the top surface of the small pieces, i.e., 10 mm. The lower bound of optimization variables does not exceed the position of the objective region, i.e., −2 mm. 

**Figure 4:**
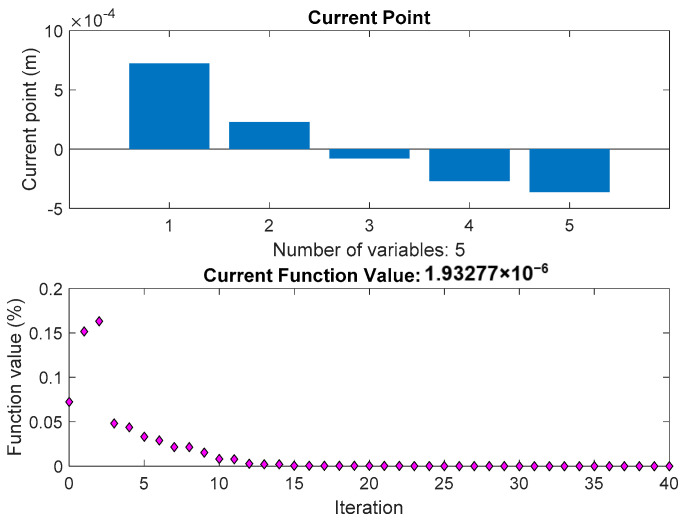
The optimization iteration and variables.

The results can be seen in Figure 4, the minimization of the objective function is obtained. In Figure 4, the ‘current point’ denotes the value of the optimization variables and is the best point the solver found in its run. The ‘current function value’ is the value of the objective function at the current point. 

**Figure 5:**
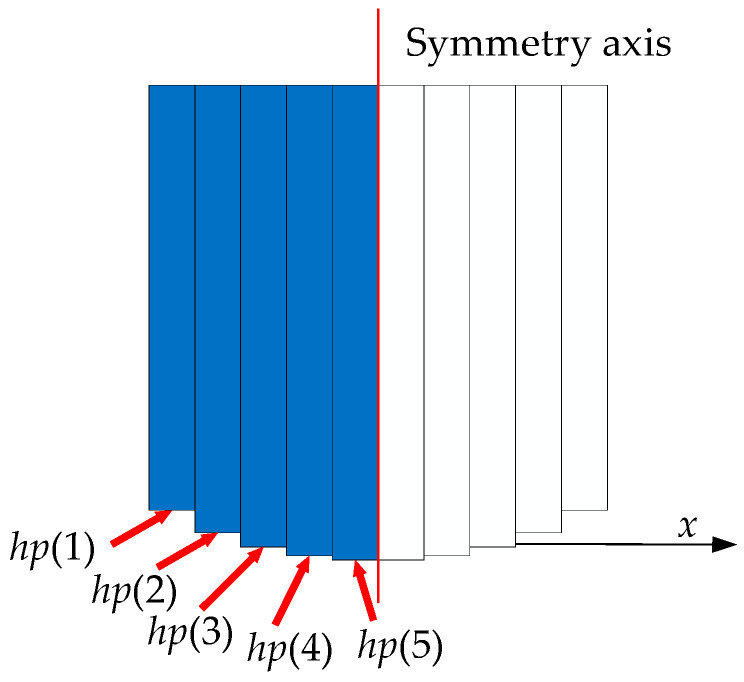
The small magnet pieces, based on the optimization variables of one permanent magnet.

The small pieces of one permanent magnet based on the optimization variables are shown in Figure 5. In the new magnet array, the optimized curved surfaces of permanent magnets in the *x* and *z* magnetization directions are the same. The symmetry axis of the curved surface is in the middle and both sides have a symmetrical distribution. *h_p_*(1)~*h_p_*(5) are the optimization variables and also the positions of bottom surfaces of small pieces of half of one permanent magnet. So the small pieces of one permanent magnet can be obtained according to symmetry. 

**Figure 6:**
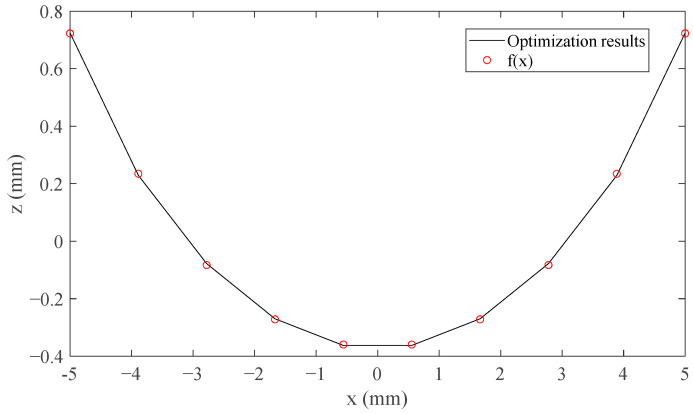
The polynomial fitting data *f*(*x*) and the optimization results for one permanent magnet.

We take the permanent magnet whose *x* coordinate of the center is at zero as an example. The shape of the optimized curved surface of the permanent magnet is shown in Figure 6. The data used to construct the curved surface shape are obtained according to the optimization results. It can be seen that the shape is similar to a sine or power function. The least squares polynomial fitting is used to fit the data. By comparing the results of a polynomial of degrees 2, 3, and 4, the fitting of the polynomial of degree 4 is more accurate. The polynomial of degree 4 is chosen. For better expression and without affecting the accuracy, the first- and third-order terms with very small coefficients are removed, and the sum of squares due to error (SSE) is 1.376 × 10^−10^ m^2^. The computed data of the polynomial is also shown in Figure 6 and the expression is as follows

(16) $$f\left(x\right)=a_{1}x^{4}+a_{2}x^{2}+a_{3}$$

where *a*_1_ = 3.763 × 10^5^, *a*_2_ = 34.28, and *a*_3_ = −3.699 × 10^−4^ are the coefficients, and the value range of *x* is [−*p*/2, *p*/2].

According to the optimization and polynomial fitting, the analytical model of the magnetic flux density is simple and expressed as Equation (10).

### 3.2. Verification with FEM

**Figure 7:**
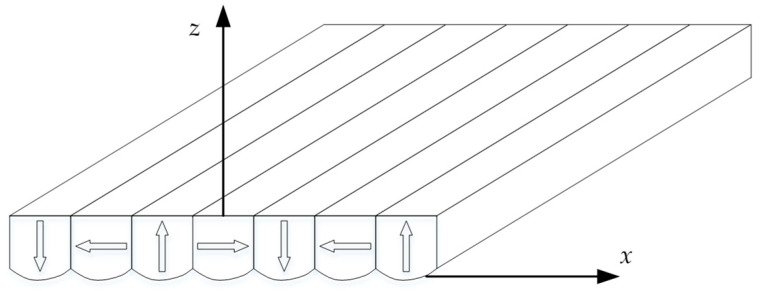
The axonometric drawing of the new magnet array with a curved surface.

The new magnet array with a curved surface is obtained by applying the optimization results, and the axonometric drawing is shown in Figure 7. In order to verify the optimization, the magnetic flux density with different *z* coordinates are compared with the finite element model (FEM). The FEM is built and analyzed by the Ansoft Maxwell. With precision-driven adaptive subdivision technology and a powerful post-processor, Ansoft Maxwell is an excellent high-performance electromagnetic design software in the industry.

**Figure 8:**
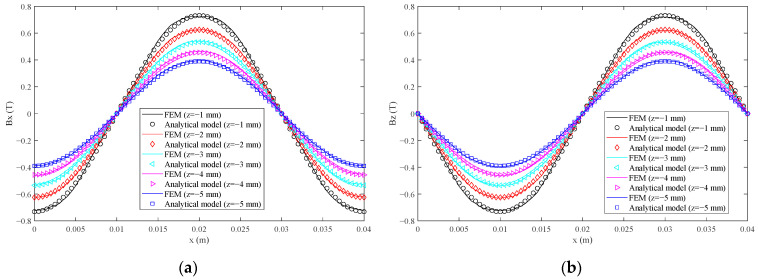
The magnetic flux density for different *z* coordinates: (**a**) the *x* component; (**b**) the *z* component.

The magnetic flux density is obtained by the FEM and analytical model. The *x* and *z* components are shown in Figure 8. It is found that the magnetic flux density has a good sinusoidal waveform. The analytical model results fit very well with the FEM results. The total harmonic distortion (THD) [23] is introduced to evaluate the magnetic flux density. The root mean square (RMS) values of the error between the analytical model and FEM and THD of magnetic flux density are shown in Table 3.

The RMS(Δ*B_x_*), RMS(Δ*B_z_*), THD(*B_x_*) and THD(*B_z_*) values for different *z* coordinates are very small. The maximum RMS(Δ*B_x_*) and RMS(Δ*B_z_*) values occurred when *z* is −1 mm, which are 2.19% and 2.14% of the peak value of *B_x_* and *B_z_* of FEM, respectively. The THD(*B_x_*) and THD(*B_z_*) values are the same according to Equation (7). The maximum THD(*B_x_*) and THD(*B_z_*) values occurred when *z* is −1 mm, which are both 2.69%. As a result, a new permanent magnet array with a good sinusoidal magnetic field and a simple analytical model of magnetic flux density is obtained.

## 4. Discussion

### 4.1. The Applicability of Optimization Results

#### 4.1.1. Different Lengths of Permanent Magnet

In this section, the applicability of optimization results will be discussed when the dimensions of the permanent magnet have changed. The optimization results are obtained with the specific parameters of the permanent magnet array. The fit expression must be improved for different dimensions of the permanent magnet, and the new expression is described as

(17) $$f_{0}\left(x_{0}\right)=\left(a_{1}x_{0}{}^{4}+a_{2}x_{0}{}^{2}+a_{3}\right)\frac{p_{1}}{p},$$

where *p*_1_ is the side length of new permanent magnets which are magnetized in *z* and *x* directions, *x*_1_ is the new *x* coordinate, and the value range is [−*p*_1_/2, *p*_1_/2],

(18) $$x_{0}=x_{1}p/p_{1}$$

**Figure 9:**
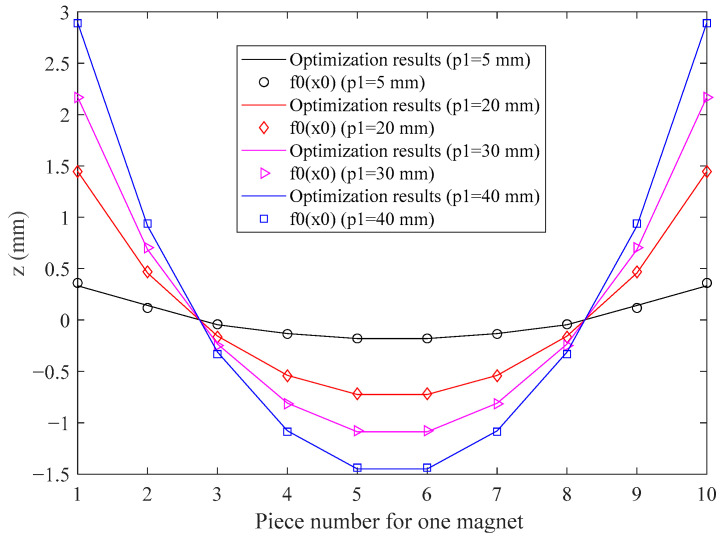
The optimization results and fitting data *f*_0_(*x*_0_) for one permanent magnet with different *p*_1_ values.

We take *p*_1_ as 5 mm, 20 mm, 30 mm, and 40 mm, respectively, as an example to verify the new fit expression. Figure 9 shows the results of optimization and the computed data of the new fit expression. It can be seen that the new fit expression has good consistency with the optimization results. Therefore, the new fit expression has good applicability to the permanent magnet with different side lengths.

#### 4.1.2. Different Heights of Permanent Magnet

**Figure 10:**
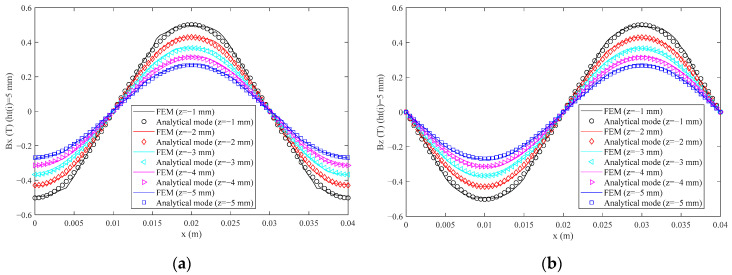
The magnetic flux density in different *z* coordinates when *h_t_*(*i*) (*i* = 1, 2, …, 5) takes 5 mm: (**a**) the *x* component; (**b**) the *z* component.

**Figure 11:**
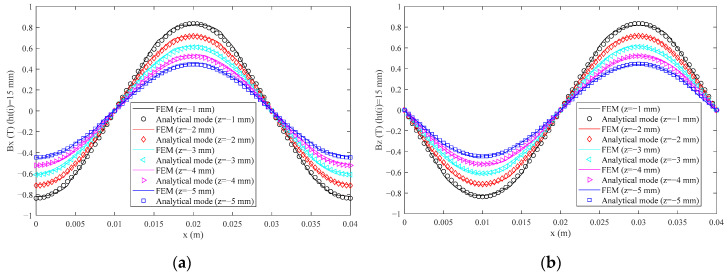
The magnetic flux density in different *z* coordinates when *h_t_*(*i*) (*i* = 1, 2, …, 5) takes 15 mm: (**a**) the *x* component; (**b**) the *z* component.

The applicability of optimization results is also analyzed when the height of the permanent magnet is changed. The magnetic flux density of the analytical model and FEM are compared when *h_t_*(*i*) (*i* = 1, 2, …, 5) takes 5 mm and 15 mm, respectively, which are shown in Figure 10 and Figure 11. 

From Figure 10 and Figure 11, the magnetic flux density also shows a good sinusoidal waveform. The analytical model results fit very well with the FEM results. The maximum RMS(Δ*B_x_*) and RMS(Δ*B_z_*) values are 0.0151 T and 0.0139 T, and 3.03% and 2.78% of the peak value of *B_x_* and *B_z_* of FEM when *h_t_*(*i*) (*i* = 1, 2, …, 5) takes 5 mm, respectively. The maximum RMS(Δ*B_x_*) and RMS(Δ*B_z_*) values are 0.0183 T and 0.0168 T, and 2.22% and 2.06% of the peak value of *B_x_* and *B_z_* of FEM when *h_t_*(*i*) (*i* = 1, 2, …, 5) takes 15 mm, respectively. The maximum THD(*B_x_*) and THD(*B_z_*) values also occur when *z* takes −1 mm, which are 3.53% and 2.37% of the peak value when *h_t_*(*i*) (*i* = 1, 2, …, 5) takes 5 mm and 15 mm respectively. Therefore, the new fit expression has good applicability to the permanent magnet with different heights. 

The applicability of optimization results is verified by analyzing the new magnet arrays with different lengths and heights of permanent magnets. Therefore, the new magnet array can be flexibly designed according to the feature. As long as the shape of the bottom curved surface is consistent with the optimization results, the desired magnetic field strength can be obtained by simply changing the size of the permanent magnet and the magnetic field will still have good sinusoidal characteristics. 

### 4.2. The Effect on the Mass

In the new magnet array, the cross-section of the permanent magnet is no longer a rectangle when the curved surface is applied. It is necessary to analyze the effect on the mass. The total mass can be obtained by summing up the mass of all permanent magnets. It is assumed that there is no difference between permanent magnets, so the effect on the mass can be indicated by analyzing one permanent magnet. By analyzing the cross-section of the permanent magnet, the change of mass can be obtained by integrating equation *f*_0_(*x*_0_). The mass decreases when the result is positive, otherwise, it increases. The integral is expressed as

(19) $$I_{f_{0}}=\int_{-\frac{p_{1}}{2}}^{\frac{p_{1}}{2}}f_{0}\left(x_{0}\right)dx_{0}=\left(3a_{1}p^{4}+20a_{2}p^{2}+240a_{3}\right)\frac{p_{1}{}^{2}}{240p}.$$

The area ratio of the cross-section between the new permanent magnet and the rectangular permanent magnet is analyzed. The area of the cross-section of the rectangular permanent magnet can be described as

(20) $$A_{c}=p_{1}\times k_{h}p_{1}=k_{h}p_{1}{}^{2},$$

where *k_h_* × *p*_1_ is the height of the cross-section and *k_h_* is the coefficient.

The ratio is obtained by

(21) $$R_{m}=\frac{I_{f_{0}}}{A_{c}}\times 100\%=\left(3a_{1}p^{4}+20a_{2}p^{2}+240a_{3}\right)\frac{5}{12pk_{h}}.$$

It is found that the ratio is independent of the parameter *p*_1_. The parameter *p* is set to 10 mm in the design of the surface. The ratio is a fixed value when *k_h_* is given. Taking *k_h_* = 1 as an example, the ratio value is −0.372%. The increase is very little and has almost no effect on the mass.

### 4.3. The Effect on the Air Gap

Due to the curved surface of permanent magnets, the bottom of the new magnet array is not flat. A part of the bottom curved surface is below the *x*-axis, according to the optimization results. Compared with the conventional magnet array, the actual air gap between the magnet and the coil is a little smaller. The magnetic field strength will be changed when the air gap remains the same as the conventional magnet array. The influence of the curved surface on the air gap is investigated further. 

The lowest point of the curved surface is obtained when *x*_0_ is 0 mm in *f*_0_(*x*_0_). To keep the same air gap, the new *z* coordinate value, at which the magnetic flux density is calculated, is

(22) $$z=z_{0}+f_{0}\left(x_{0}=0\right)=z_{0}+\frac{a_{3}p_{1}}{p},$$

where *z*_0_ is the original coordinate value, such as −1 mm, or −2 mm, and so on.

The magnetic flux density is proportional to the expression of *e^ωz^* according to Equation (10). The ratio of the magnetic flux density calculated at the new *z* coordinate value to the original one is expressed as

(23) $$R_{z}=e^{\omega_{1}z}/e^{\omega_{1}z_{0}}\times 100\%,$$

where *ω*_1_ is redefined for different side lengths of the permanent magnet,

(24) $$\omega_{1}=\frac{\pi }{2p_{1}}.$$

The ratio is also a fixed value and independent of the parameter *p*_1_, which equals *e^π/2/p×a^_3_*. The value of the ratio is 94.36% for different lengths of the permanent magnet. Considering the air gap, the magnetic flux density is slightly decreased. In other words, the new magnet array has a good sinusoidal magnetic field in a very small air gap, sacrificing very little magnetic flux density, which is especially suitable for precision positioning apparatus with strict requirements for a small air gap.

### 4.4. The Effect on the Force 

In order to investigate the effect on the force of the new magnet array, the force and force ripple of the new magnet array and the conventional magnet array are compared. In the analytical model for real-time control, the expressions of the magnetic flux density of the two magnet arrays are the same and obtained by Equation (10). 

**Figure 12:**
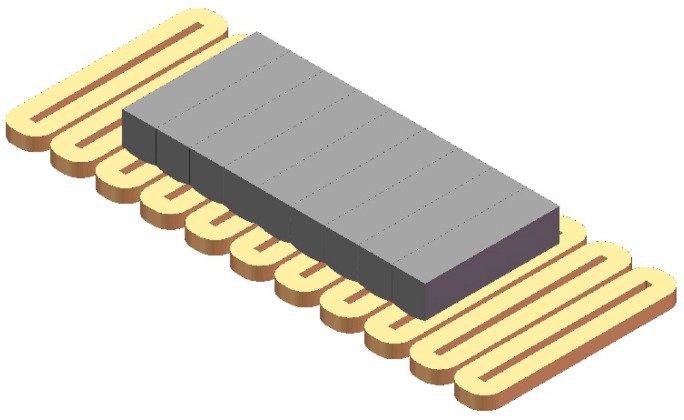
An axonometric diagram of the motor with the new magnet array and the three-phase coil.

We take the linear motor with a moving magnet array as an example. Figure 12 shows the axonometric diagram of the motor with the new magnet array and coils. The motor with the conventional magnet array is similar and the diagram is not given.

**Figure 13:**
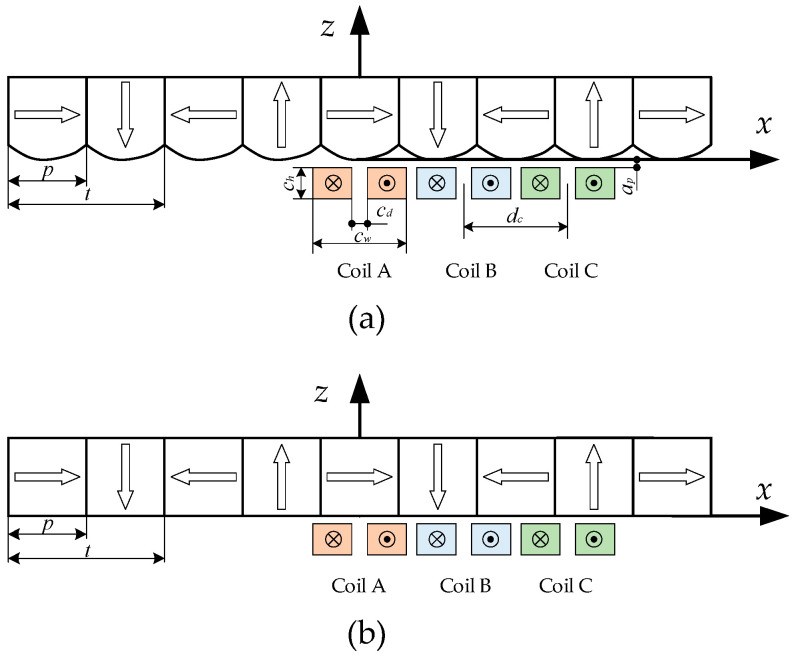
A cross-section of the motor with three-phase coils: (**a**) the new magnet array; (**b**) the conventional magnet array.

For the convenience of analysis, Figure 13 shows the cross-section of the motor with the new magnet array and the conventional magnet array. In order to remove the position dependency and generate the position-independent force on the array, the *dq*0 transformation and the three-phase coil are applied.

By using the magnetic flux density of Equation (10), the Lorentz force on the magnet array, generated by one coil, is calculated by solving the volume integral and expressed as

(25) $$\left[\begin{array}{c} F_{cx}\\ F_{cz} \end{array}\right]=NIK_{F}\left(e^{\omega c_{t}}-e^{\omega c_{b}}\right)\left[\begin{array}{c} \cos\left(\omega x_{co}\right)\\ \sin\left(\omega x_{co}\right) \end{array}\right],$$

where

(26) $$K_{F}=\frac{8m_{l}K_{1}}{c_{h}\left(c_{w}-c_{d}\right)\omega^{2}}\sin\frac{\omega \left(c_{d}+c_{w}\right)}{4}\sin\frac{\omega \left(c_{w}-c_{d}\right)}{4}$$

(27) $$c_{h}=c_{t}-c_{b}$$

where *N* is the number of coil turns, *I* is the current in the coil, *K_F_* is the coefficient, *c_t_* and *c_b_* are the position of the top and bottom surfaces of the coil, respectively. *x_co_* is the coordinate of the coil center in the *x*-direction, *m_l_* is the length of the magnet array in the *y*-direction, *c_d_* is the width of the air core of the coil, *c_w_* is the width of the coil, and *c_h_* is the height of the coil. 

For the three-phase coil group, the force can be expressed as

(28) $$\begin{array}{l} \vec{F}=\left[\begin{array}{c} F_{x}\\ F_{z} \end{array}\right]=NK_{F}\left(e^{\omega c_{t}}-e^{\omega c_{b}}\right)T_{F}\vec{i}\\ =NK_{F}\left(e^{\omega c_{t}}-e^{\omega c_{b}}\right)\left[\begin{array}{ccc} \cos\left(\omega x_{a}\right) & \cos\left(\omega x_{b}\right) & \cos\left(\omega x_{c}\right)\\ \sin\left(\omega x_{a}\right) & \sin\left(\omega x_{b}\right) & \sin\left(\omega x_{c}\right) \end{array}\right]\vec{i} \end{array}$$

where

(29) $$\vec{i}={\left[\begin{array}{ccc} i_{a} & i_{b} & i_{c} \end{array}\right]}^{T}$$

(30) $$x_{b}=x_{a}+d_{c}$$

(31) $$x_{c}=x_{a}+2d_{c}$$

(32) $$d_{c}=\frac{2t}{3}$$

where *T_F_* is the matrix, *x_a_*, *x_b,_* and *x_c_* are the coordinates of the coil center of the three-phase coil in the *x*-direction, and *d_c_* is the distance between two coil centers. 

The *dq*0 transformation matrix is used to remove the position dependency and expressed as

(33) $$T_{dq0}=\frac{2}{3}\left[\begin{array}{ccc} \cos\left(\omega x\right) & \cos\left(\omega x+\frac{2}{3}\pi \right) & \cos\left(\omega x-\frac{2}{3}\pi \right)\\ -\sin\left(\omega x\right) & -\sin\left(\omega x+\frac{2}{3}\pi \right) & -\sin\left(\omega x-\frac{2}{3}\pi \right)\\ \frac{1}{2} & \frac{1}{2} & \frac{1}{2} \end{array}\right]$$

By applying the *dq*0 transformation matrix, the force of the three-phase coil group is replaced as

(34) $$\vec{F}=\left[\begin{array}{c} F_{x}\\ F_{z} \end{array}\right]=NK_{F}\left(e^{\omega c_{t}}-e^{\omega c_{b}}\right)T_{F}T_{dq0}^{-1}\left[\begin{array}{c} i_{d}\\ i_{q}\\ 0 \end{array}\right]=\frac{3}{2}NK_{F}\left(e^{\omega c_{t}}-e^{\omega c_{b}}\right)\left[\begin{array}{c} i_{d}\\ -i_{q} \end{array}\right]$$

So the current of the coil is expressed as

(35) $$\vec{i}=\left[\begin{array}{c} i_{a}\\ i_{b}\\ i_{c} \end{array}\right]=T_{dq0}^{-1}\left[\begin{array}{c} i_{d}\\ i_{q}\\ 0 \end{array}\right]$$

From Equation (34), the levitation force and horizontal thrust are produced by the *d* axis and *q* axis current, respectively. Therefore, the position-independent force on the array generated by the three-phase coil is obtained. 

The force and force ripple of the two magnet arrays are analyzed and compared. The dimensions of the magnet array shown in Table 1 are used, and the other dimensions of the motor are given in Table 4.

**Figure 14:**
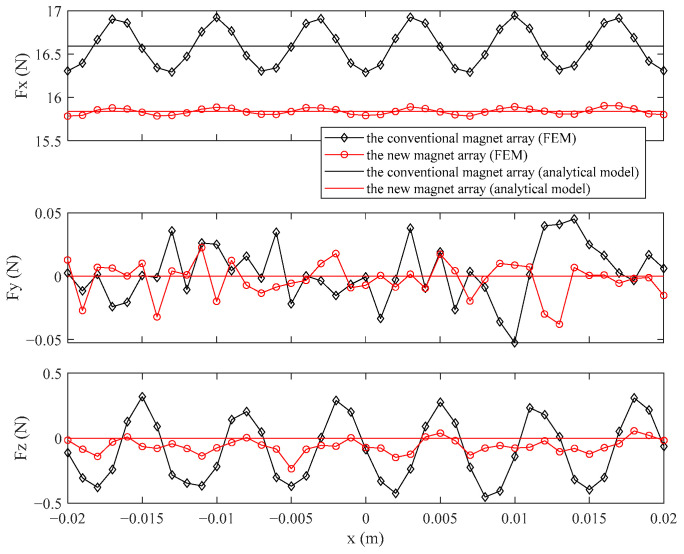
The force calculated by the FEM, the analytical model of the new magnet array, and the conventional magnet array in *x*, *y*, and *z* directions.

The force of the analytical model can be directly calculated by Equation (34) when *i_d_* and *i_q_* are given. In order to better analyze the effect on the force of the two magnet arrays, the forces of the two magnet arrays calculated by the FEM and analytical model are shown in Figure 14 when the magnet array moves in one period. The *i_d_* and *i_q_* take −5 A and 0 A respectively. 

It is found that the *F_x_* ripple and *F_z_* ripple of the new magnet array are much smaller than the conventional magnet array. The disturbing force, *F_y_*, is produced by the end of the coil due to the end effect of magnetic flux density according to the Lorentz force formula. The *F_y_* ripple for both magnet arrays is small and essentially zero. The *F_x_* of the new magnet array is slightly smaller than the force of the conventional magnet array which can be inferred from the above analysis of the effect on the air gap. 

The new magnet array is built based on the fitting curve, and the magnetic flux density of the new magnet array is expressed in Equation (10), based on the analysis in Section 3. It can be seen from Figure 7 that the magnetic flux density in different *z* coordinates has a very small error between the analytical model and FEM, but the accumulative error will be produced in the force calculation due to the volume integral by using the analytical model. Therefore, the correction coefficient, *K_c_*, is introduced to establish the analytical model of force for the new magnet array. The force of the new magnet array is modified as follows

(36) $${\vec{F}}_{new}=\left[\begin{array}{c} F_{xnew}\\ F_{znew} \end{array}\right]=\frac{3}{2}NK_{c}K_{F}\left(e^{\omega c_{t}}-e^{\omega c_{b}}\right)\left[\begin{array}{c} i_{d}\\ -i_{q} \end{array}\right]$$

where

(37) $$K_{c}=\frac{{\bar{F}}_{xFEM}}{F_{x}}$$

where *F_x_* is the *x* component of the force obtained by Equation (34), ${\bar{F}}_{xFEM}$ is the average value of the *x* component of the force obtained by the FEM in one period. 

The force of the analytical model and RMS values of the error between the analytical model and FEM for the two magnet arrays are shown in Table 5. 

From Table 5, the *F_x_* value of the new magnet array is 95.47% of the conventional magnet array. The RMS(∆*F_y_*) for the two magnet arrays are all very small. The RMS(∆*F_x_*) and RMS(∆*F_z_*) values of the new magnet array are reduced a lot, which are 16.01% and 31.75% of the conventional magnet array. Therefore, the new magnet array can significantly reduce the force ripple without sacrificing too much force in comparison to the conventional magnet array.

**Figure 15:**
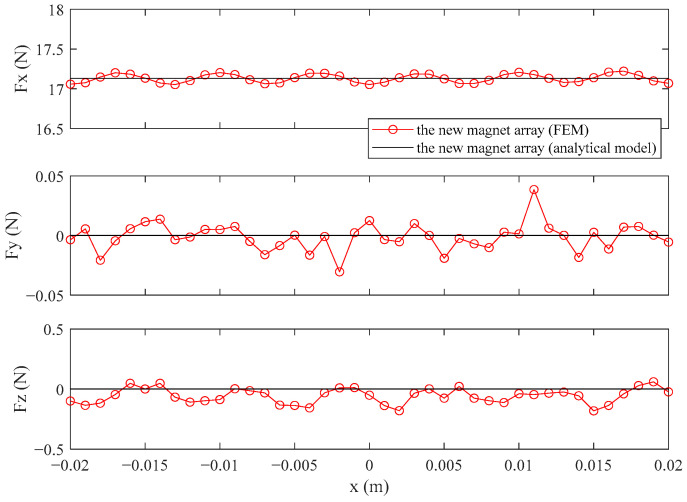
The force of the new magnet array calculated by the FEM and analytical model, based on the new air gap.

In order to better observe the advantages of the new magnet array in the small air gap and verify the applicability of the correction coefficient, the air gap, *a_p_*, is changed from 1 mm to 0.5 mm. The force of the new magnet array calculated by the FEM and analytical model based on the new air gap is shown in Figure 15. The force of the analytical model and RMS values of the error between the analytical model and FEM for the new magnet array based on the new air gap is shown in Table 6. 

It is found that the new magnet array still has a small force ripple with the small air gap from Figure 15. The correction coefficient is verified by analyzing the RMS values of the error between the analytical model and FEM from Table 6. The RMS(∆*F_y_*) of the new magnet array is also very small. Compared with the conventional magnet array, with the air gap of 1 mm, the force of the new magnet array is higher and the RMS(∆*F_x_*) and RMS(∆*F_z_*) values are also relatively low, which are 23.11% and 32.82% of the conventional magnet array.

To sum up, through the analysis of the above discussion, the new magnet array with a sinusoidal magnetic field is obtained. The curved surface based on the optimization results has good applicability to different dimensions of the permanent magnet. The new magnet array can be designed flexibly according to the feature. In contrast to the conventional magnet array, the new magnet array has little influence on the mass and air gap and can significantly reduce the force ripple. The new magnet array still maintains the small force ripple in the small air gap. 

## 5. Conclusions

A new 1D Halbach magnet array with a curved surface of the permanent magnet is proposed in this paper. The curved surface design method of the permanent magnet is realized based on the superposition principle. The shape of the optimized curved surface is similar to a sine or power function and fitted by a polynomial. The expression of magnetic flux density is simple and the same as the first harmonic of the conventional magnet array with rectangular permanent magnets. A good sinusoidal magnetic field is obtained in a very small air gap. The optimization results have good applicability to different dimensions of the permanent magnet. The effect on the mass of the permanent magnet array is very small. The magnetic flux density is slightly decreased when the air gap remains the same as the conventional magnet array. The new magnet array can significantly reduce the force ripple in comparison to the conventional magnet array. The new magnet array is especially suitable for precision positioning apparatus with strict requirements for a small air gap. The method of surface design of permanent magnet can be used for similar 2D permanent magnet arrays.

In future work, a combined global-local optimization method will be further investigated to improve the optimization and identify the exact solution. Furthermore, the model structural design methodology will be studied to determine the fitted terms automatically.

## Figures and Tables

**Table 1:** Optimization parameters.

Parameters	Symbol	Value	Unit
pole pitch	*t*	20	mm
the side length of the magnets	*p*	10	mm
remanence of the permanent magnets	*B_r_*	1.2	T
the number of pieces of half of one permanent magnet	*n*	5	-
the positions of the top surface of each piece	*h_t_*(1)*~h_t_*(5)	10	mm

**Table 2:** Optimization variables.

Optimization variables	Constraints	Unit
*h_p_*(1)~*h_p_*(5)	[−2,10]	mm

**Table 3:** The RMS values of the error between the analytical model and FEM and THD of magnetic flux density.

*z* (mm)	RMS (∆*B_x_*) (T)	Ratio of Peak Value of *B_x_* of FEM (%)	THD (*B_x_*) (%)	RMS (∆*B_z_*) (T)	Ratio of Peak Value of *B_z_* of FEM (%)	THD (*B_z_*) (%)
−1	0.0159	2.19	2.69	0.0154	2.14	2.69
−2	0.0094	1.50	1.43	0.0075	1.20	1.43
−3	0.0075	1.40	0.76	0.0058	1.08	0.76
−4	0.0063	1.38	0.41	0.0059	1.28	0.41
−5	0.0060	1.52	0.22	0.0051	1.29	0.22

**Table 4:** The dimensions of the motor.

Parameters	Symbol	Value	Unit
air gap	*a_p_*	1	mm
the length of the magnet array in the *y*-direction	*m_l_*	40	mm
the height of the coil	*c_h_*	4	mm
the width of the coil	*c_w_*	12	mm
the width of the air core of the coil	*c_d_*	2	mm
the distance between two coil centers	*d_c_*	13.33	mm
the number of coil turns	*N*	100	-

**Table 5:** The analytical force and RMS values for the two magnet arrays.

Magnet Array	*F_x_* (N)	RMS (∆*F_x_*)(N)	*F_y_* (N)	RMS (∆*F_y_*) (N)	*F_z_* (N)	RMS (∆*F_z_*) (N)
**The** **conventional magnet array**	16.5901	0.2280	0	0.0244	0	0.2611
**The new** **magnet array**	15.8390	0.0365	0	0.0138	0	0.0829

**Table 6:** The analytical force and RMS values for the new magnet array, based on the new air gap.

Magnet Array	*F_x_*(N)	RMS(∆*F_x_*)(N)	*F_y_*(N)	RMS(∆*F_y_*) (N)	*F_z_*(N)	RMS(∆*F_z_*) (N)
**The new magnet array**	17.1322	0.0527	0	0.0116	0	0.0857

## Data Availability

The data presented in this study are available in the supplementary material, Data.zip.

## Supplementary Materials

The following are available online at https://www.mdpi.com/article/10.3390/s21072522/s1.

## References

[1] Rahideh A., Ghaffari A., Barzegar A., Mahmoudi A. (2018). Analytical Model of Slotless Brushless PM Linear Motors Considering Different Magnetization Patterns. IEEE Trans. Energy Conver..

[2] Kim W.J., Trumper D.L. (1998). High-precision magnetic levitation stage for photolithography. Precis. Eng..

[3] Guo L., Zhang H., Galea M., Li J., Gerada C. (2016). Multiobjective Optimization of a Magnetically Levitated Planar Motor with Multilayer Windings. IEEE Trans. Ind. Electron..

[4] Wang Y., Chen X., Luo X., Zeng L. (2018). Analysis and Optimization of a Novel 2-D Magnet Array with Gaps and Staggers for a Moving-Magnet Planar Motor. Sensors.

[5] Xu F., Lv Y., Xu X., Dinavahi V. (2018). FPGA-Based Real-Time Wrench Model of Direct Current Driven Magnetic Levitation Actuator. IEEE Trans. Ind. Electron..

[6] Yoon J.Y., Lang J.H., Trumper D.L. (2019). Double-Sided Linear Iron-Core Fine-Tooth Motor for Low Acoustic Noise and High Acceleration. IEEE-ASME Trans. Mech..

[7] Chi S., Yan J., Shan L., Wang P. (2019). Detent Force Minimizing for Moving-Magnet-Type Linear Synchronous Motor. IEEE Trans. Magn..

[8] Eckert P.R., Flores Filho A.F., Perondi E., Ferri J., Goltz E. (2016). Design Methodology of a Dual-Halbach Array Linear Actuator with Thermal-Electromagnetic Coupling. Sensors.

[9] Trumper D.L., Kim W.J., Williams M.E. (1996). Design and analysis framework for linear permanent-magnet machines. IEEE Trans. Ind. Appl..

[10] Kim W.J., Trumper D.L., Lang J.H. (1998). Modeling and vector control of planar magnetic levitator. IEEE Trans. Ind. Appl..

[11] Lee M.G., Gweon D.G. (2004). Optimal design of a double-sided linear motor with a multi-segmented trapezoidal magnet array for a high precision positioning system. J. Magn. Magn. Mater..

[12] Chen J., Zhang B., Ding Y., Ding H. (2016). Field analysis of a sinusoidal-edged Halbach magnet array using the differential quadrature finite element method. Int. J. Appl. Electrom..

[13] Usman I.U.R., Lu X. (2015). Force Ripple Attenuation of 6-DOF Direct Drive Permanent Magnet Planar Levitating Synchronous Motors. IEEE Trans. Magn..

[14] Rui C. (2016). A New Type of Magnet Array for Planar Motor. Master’s Thesis.

[15] Boduroglu A., Gulec M., Demir Y., Yolacan E., Aydin M. (2019). A New Asymmetric Planar V-Shaped Magnet Arrangement for A Linear PM Synchronous Motor. IEEE Trans. Magn..

[16] Zhang Y., Martínez-García M., Kalawsky R.S., Latimer A. (2020). Grey-box Modelling of the Swirl Characteristics in Gas Turbine Combustion System. Measurement.

[17] Mai H.C.M., Dubas F., Chamagne D., Espanet C. Optimal design of a surface mounted permanent magnet in-wheel motor for an urban hybrid vehicle. Proceedings of the IEEE Vehicle Power and Propulsion Conference.

[18] Dong H.K., Hirota K. (2008). Vector control for loss minimization of induction motor using GA–PSO. Appl. Soft. Comput..

[19] Gangl P., Amstutz S., Langer U. (2015). Topology Optimization of Electric Motor Using Topological Derivative for Nonlinear Magnetostatics. IEEE Trans. Magn..

[20] Shiri A., Shoulaie A. (2012). Design Optimization and Analysis of Single-Sided Linear Induction Motor, Considering All Phenomena. IEEE Trans. Energy Conver..

[21] Zhang Y., Martínez-García M., Serrano-Cruz J.R., Latimer A. Multi-region System Modelling by using Genetic Programming to Extract Rule Consequent Functions in a TSK Fuzzy System. Proceedings of the IEEE 4th International Conference on Advanced Robotics and Mechatronics (ICARM).

[22] Xue X.D., Cheng K.W.E., Ho S.L., Sutanto D. Precise analytical modelling magnetic characteristics of switched reluctance motor drives using two-dimensional least squares. Proceedings of the IEEE 34th Annual Conference on Power Electronics Specialist.

[23] Milan S., Aidar Z., Tohid A., Yakov L., Gabriele G., Alex R. (2017). Simultaneous Selective Harmonic Elimination and THD Minimization for a Single-Phase Multilevel Inverter with Staircase Modulation. IEEE Trans. Ind. Appl..

